# An unusual cause of free air in the abdomen: emphysematous cystitis with bladder diverticulum perforation

**DOI:** 10.1259/bjrcr.20210126

**Published:** 2022-03-09

**Authors:** Archie G M Keeling, William P N Southwell, Dean Y Huang, Azhar Khan

**Affiliations:** 1 King’s College Hospital, London, United Kingdom; 2 King’s College, London, United Kingdom

## Abstract

A 64-year-old male, with a history of chronic urinary outflow obstruction secondary to benign prostatic hyperplasia, presented with haematuria and urinary retention following spontaneous removal of his long-term catheter. The patient was septic on admission and a CT examination of the abdomen and pelvis showed an acutely inflamed urinary bladder diverticulum and extensive intra-abdominal free air. The patient was treated medically for emphysematous cystitis centred on a perforated bladder diverticulum, which was thought to be caused by the underlying infectious/inflammatory process. Alternative aetiologies for free air in the abdomen such a traumatic bladder perforation and gastrointestinal perforation were considered and excluded. The patient responded well to medical management and was discharged after an 11 day in-patient stay.

## Background

Emphysematous cystitis is a rare but potentially fatal condition characterised by gas within the bladder wall and lumen; it has an associated mortality of 7%.^1^ Risk factors of emphysematous cystitis include diabetes mellitus, advanced age, alcoholism, urinary stasis and female sex.^2^ Causative organisms include *Escherichia coli* (58%), *Klebsiella pneumoniae* (21%), *Clostridium* spp (7%) and *Enterobacter* spp (7%).^3^ Bladder wall perforation associated with emphysematous cystitis is an extremely rare and potentially life-threatening complication and may be caused by the weakening of the bladder wall due to infectious/inflammatory processes.^4^ CT is key in establishing disease severity and determining the presence of possible complications such as ascending infection and perforation. Early diagnosis and medical management are essential in reducing potential morbidity and mortality, which may also prevent the need for surgical intervention.^5^ Surgical management may be indicated if medical therapy fails; surgical options may include nephrostomy insertion in ascending and obstructing infection, surgical debridement and partial or complete cystectomy.^6^ In this case report, we describe a case of bladder diverticulum perforating due to coincident emphysematous cystitis as an unusual cause of free air in the abdomen and how this condition could be conservatively managed effectively.

## Case presentation

A 64-year-old male patient presented to an emergency department with haematuria and urinary retention. He had been previously using a long-term catheter for benign prostatic hyperplasia, for which he had been awaiting an elective prostatectomy. However, the long-term catheter fell out 2 days before his presentation. At the time of his presentation, the patient had not passed any urine for 24 h.

His medical history included benign prostatic hypertrophy and hypertension; his medication history included ramipril. The patient lived with his son, was an ex-smoker and did not consume alcohol. On examination, the patient was diffusely tender in his lower abdomen, he had an enlarged prostate on digital rectal examination, and he had a low-grade temperature of 37.8°C.

## Investigations

At the time of the patient’s admission, blood results showed raised inflammatory markers (C-reactive protein: 179, white cell count: 8.5, neutrophils: 7.3) and renal function consistent with an acute kidney injury (urea 13.9, creatinine 222). Blood results also showed a raised glucose of 14 mmol l^−1^, however, the patient had no known diagnosis of diabetes mellitus; subsequent HBA1c measurement was shown to be elevated at 7.8%. Urine dip analysis confirmed the presence of blood, nitrates and white blood cells within his urine. When the patient became pyrexic, blood cultures were sent but these demonstrated no significant growth after 5 days. A urine sample was also sent on admission, which showed the presence of pus cells and red blood cells with “heavy mixed growth” but unfortunately no definitive causative organism was identified.

**Figure 1.**
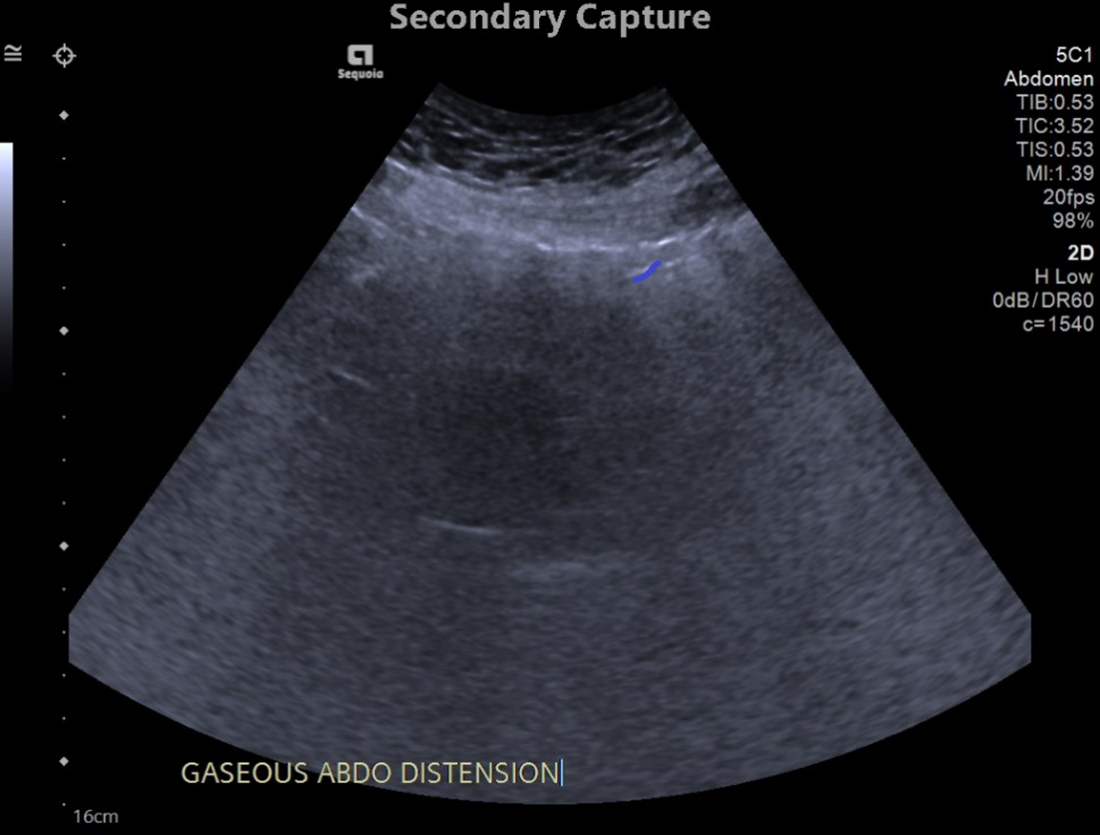
Ultrasound image of the pelvis/lower abdomen (Day 1 of admission) in the transverse orientation demonstrating a hypoechoic structure, compatible with the bladder, and with surrounding heterogenous echogenicities compatible with gas; a distinct structure in keeping with the proven bladder diverticulum was not clearly seen on this study.

**Figure 2.**
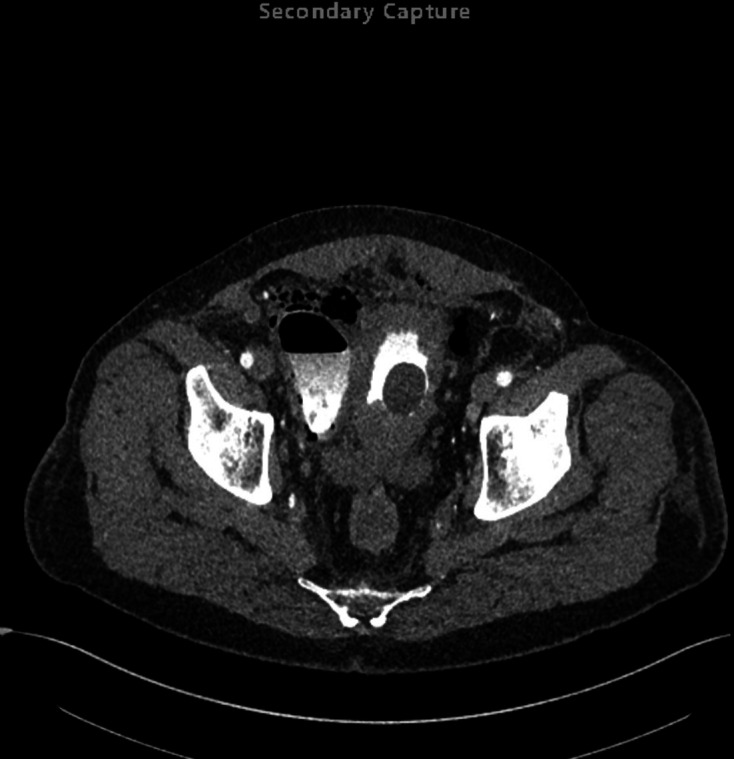
First axial delayed phase CT (Day 3 of admission), on soft tissue window setting, demonstrating a central thick-walled bladder with a Foley catheter balloon *in situ*; to the anatomical right side of the bladder there is a large bladder diverticulum containing a gas–fluid level with intramural gas; extraluminal gas is seen in the anterior antidependent regions of the pelvis/lower abdomen indicative of perforation.

**Figure 3.**
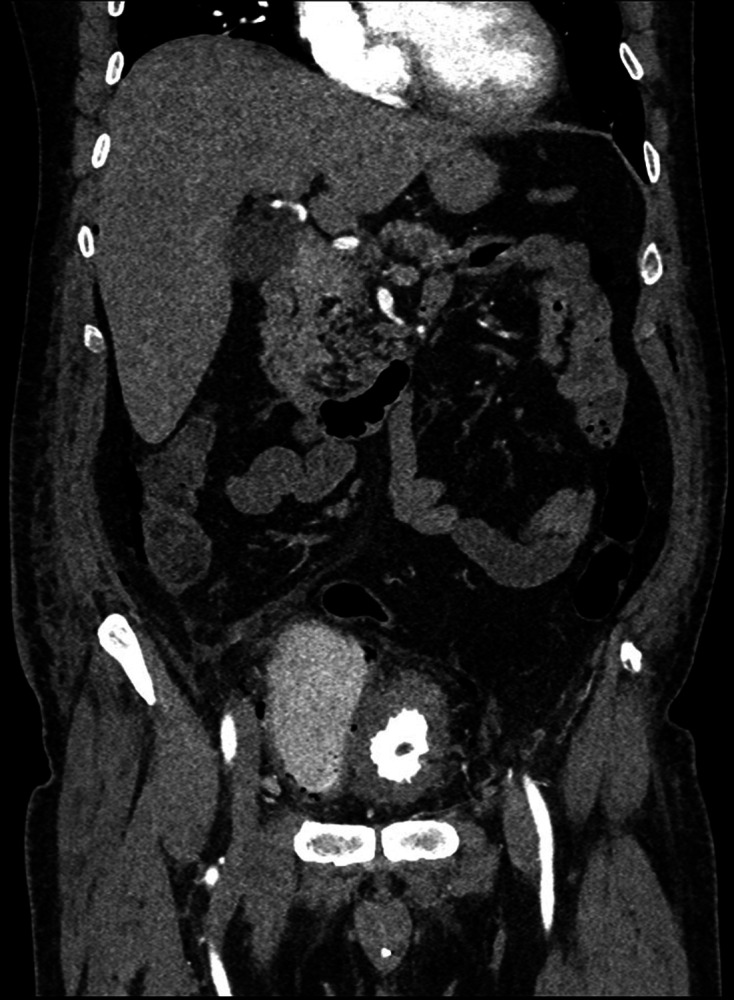
First coronal delayed phase CT (Day 3 of admission), on soft tissue window setting, demonstrating a central thick-walled bladder; to the anatomical right side of the bladder there is a large bladder diverticulum with intramural gas; extraluminal gas is seen in the right paracolic gutter indicative of perforation.

**Figure 4.**
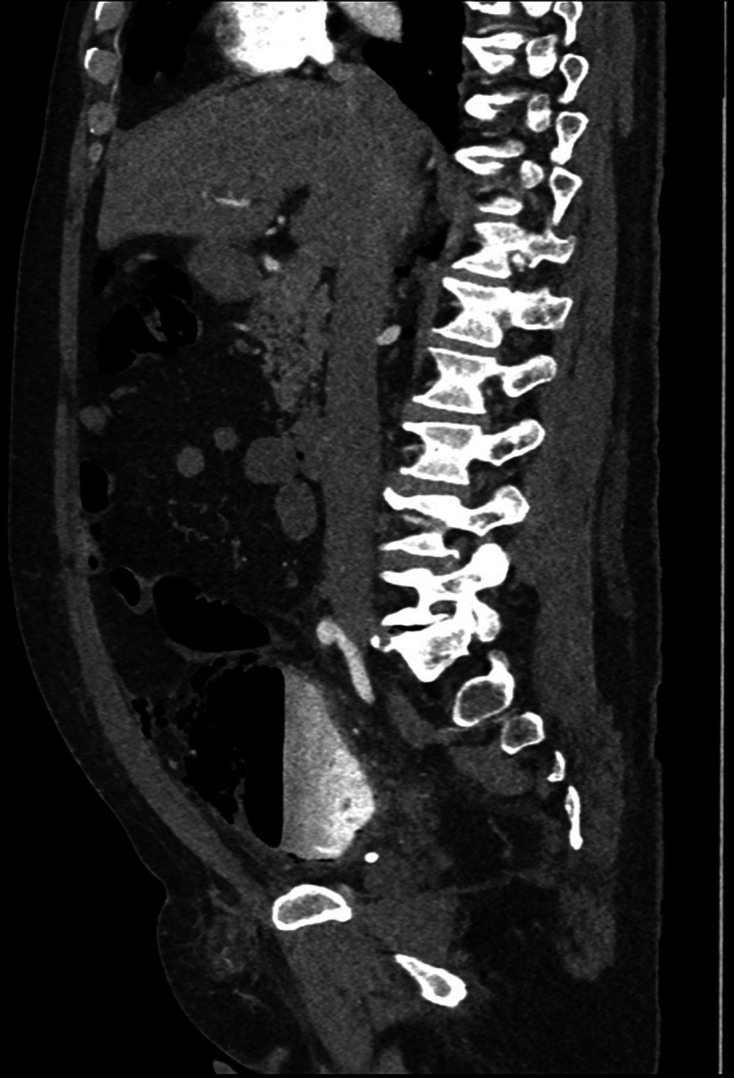
First sagittal delayed phase CT (day 3 of admission), on soft tissue window setting, demonstrating large bladder diverticulum containing a gas–fluid level with intramural gas; extraluminal gas is seen in the anterior anti dependent regions of the pelvis/lower abdomen indicative of perforation.

**Figure 5.**
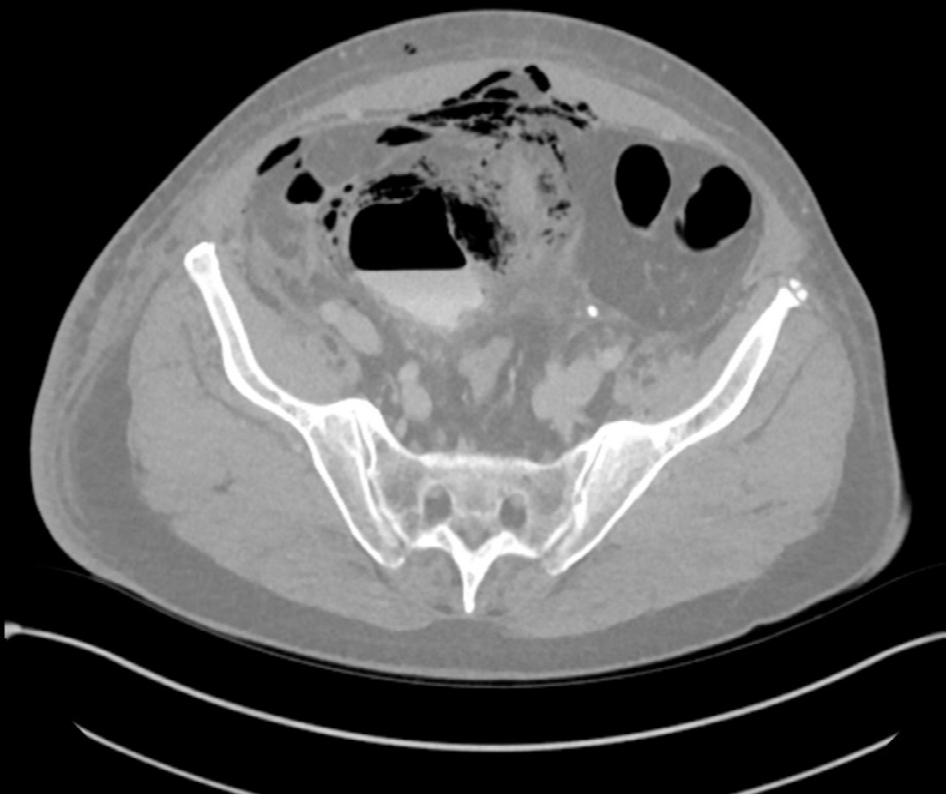
First axial delayed phase CT (Day 3 of admission), on lung window setting, highlighting the presence of extraluminal gas in the anterior anti dependent regions of the pelvis/lower abdomen, which is centred around the perforated right-sided bladder diverticulum.

**Figure 6.**
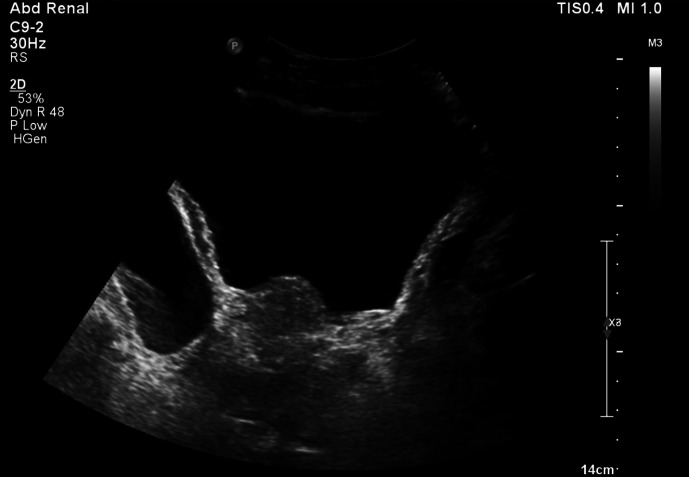
Ultrasound image of the pelvis/lower abdomen (performed prior to admission) in the transverse orientation demonstrating a central bladder; to the anatomical right of the bladder is a further hypoechoic structure compatible with a bladder diverticulum.

**Figure 7.**
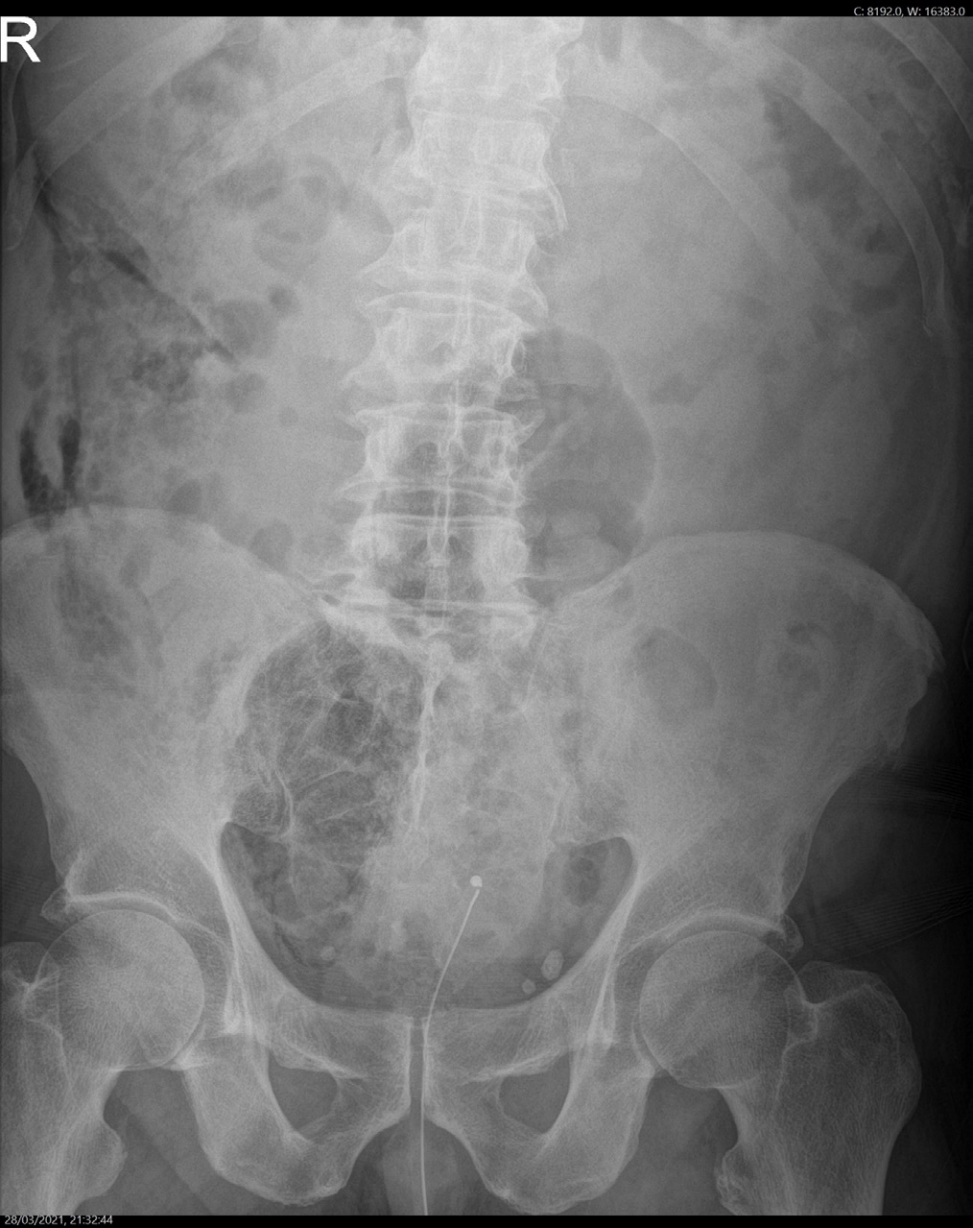
Plain abdominal radiograph shows an apparently normal bowel gas pattern, however in retrospect non-anatomical extraperitoneal free gas is seen in the right flank and in the right hemipelvis, which correlates with the subsequent CT findings.

As part of the patient’s acute kidney injury workup, an ultrasound was performed to exclude an obstructive cause (Figure 1); while there was no evidence of hydronephrosis, a 2.7 cm renal cyst was incidentally detected. Subsequently, on Day 3 of admission, a CT was performed (Figures 2–5) to further characterise this lesion; the renal cyst showed benign features, however, the presence of extensive extraperitoneal free gas was demonstrated and a urinary bladder diverticulum showed focal wall thickening with localised perilesional fat stranding, indicating acute inflammation, as well as intramural gas. The bladder diverticulum had been previously described on a historic ultrasound scan (Figure 6). Despite these acute findings, the patient remained clinically well. In retrospect, with knowledge of these CT findings, a plain abdominal radiograph (Figure 7) performed at admission showed evidence of non-anatomical extraperitoneal free air but this was mistaken for bowel gas at the time.

**Figure 8.**
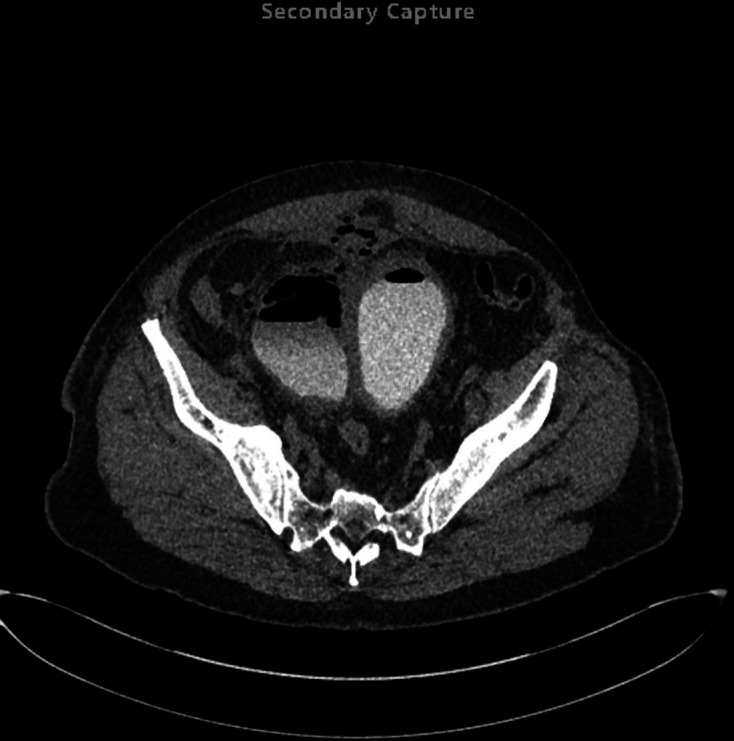
Second axial delayed phase CT (Day 8 of admission), on soft tissue window setting, demonstrating a central thick-walled bladder with intraluminal gas compatible with recent instrumentation; to the anatomical right side of the bladder there is a large bladder diverticulum containing a gas–fluid level, however, the previously demonstrated intramural gas has resolved; extraluminal gas is seen in the anterior antidependent regions of the pelvis/lower abdomen indicative of perforation, the volume of which has reduced compared to the earlier CT examination.

**Figure 9.**
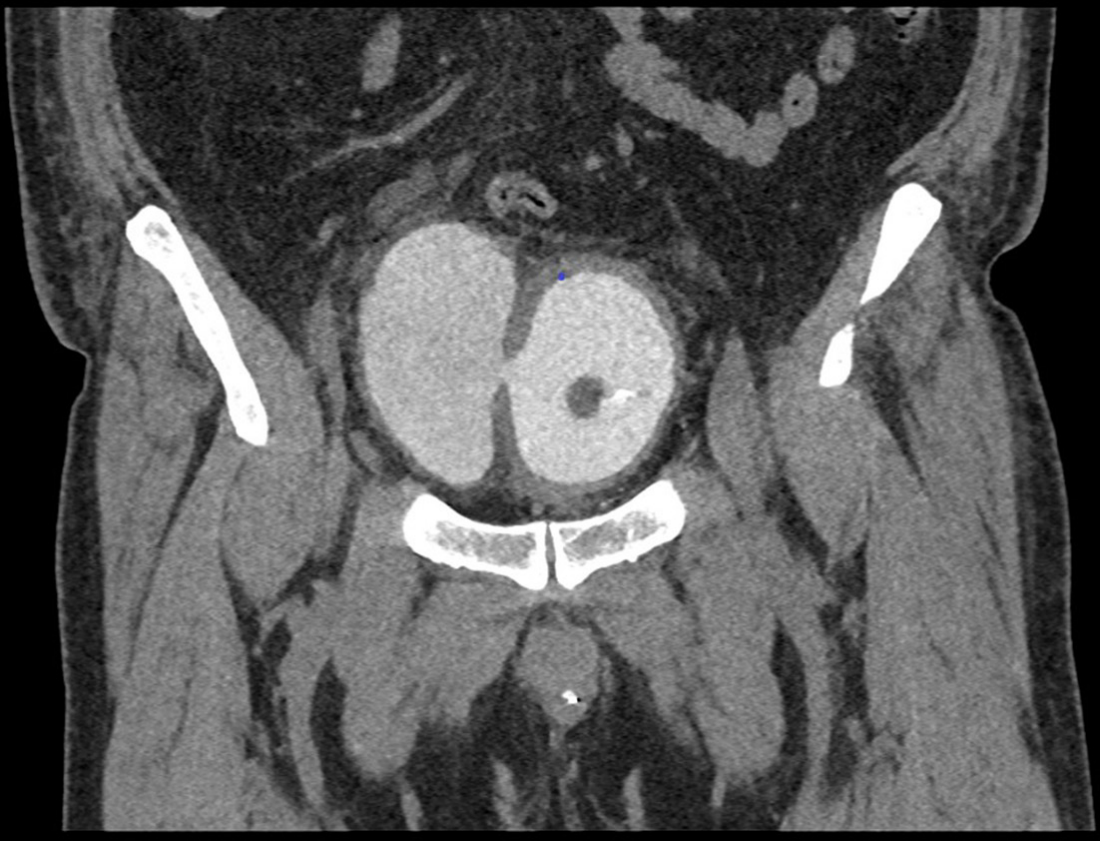
Second coronal delayed phase CT (Day 8 of admission), on soft tissue window setting, demonstrating a central thick-walled bladder; to the anatomical right side of the bladder there is a large bladder diverticulum containing a gas–fluid level however the previously demonstrated intramural gas has resolved.

**Figure 10.**
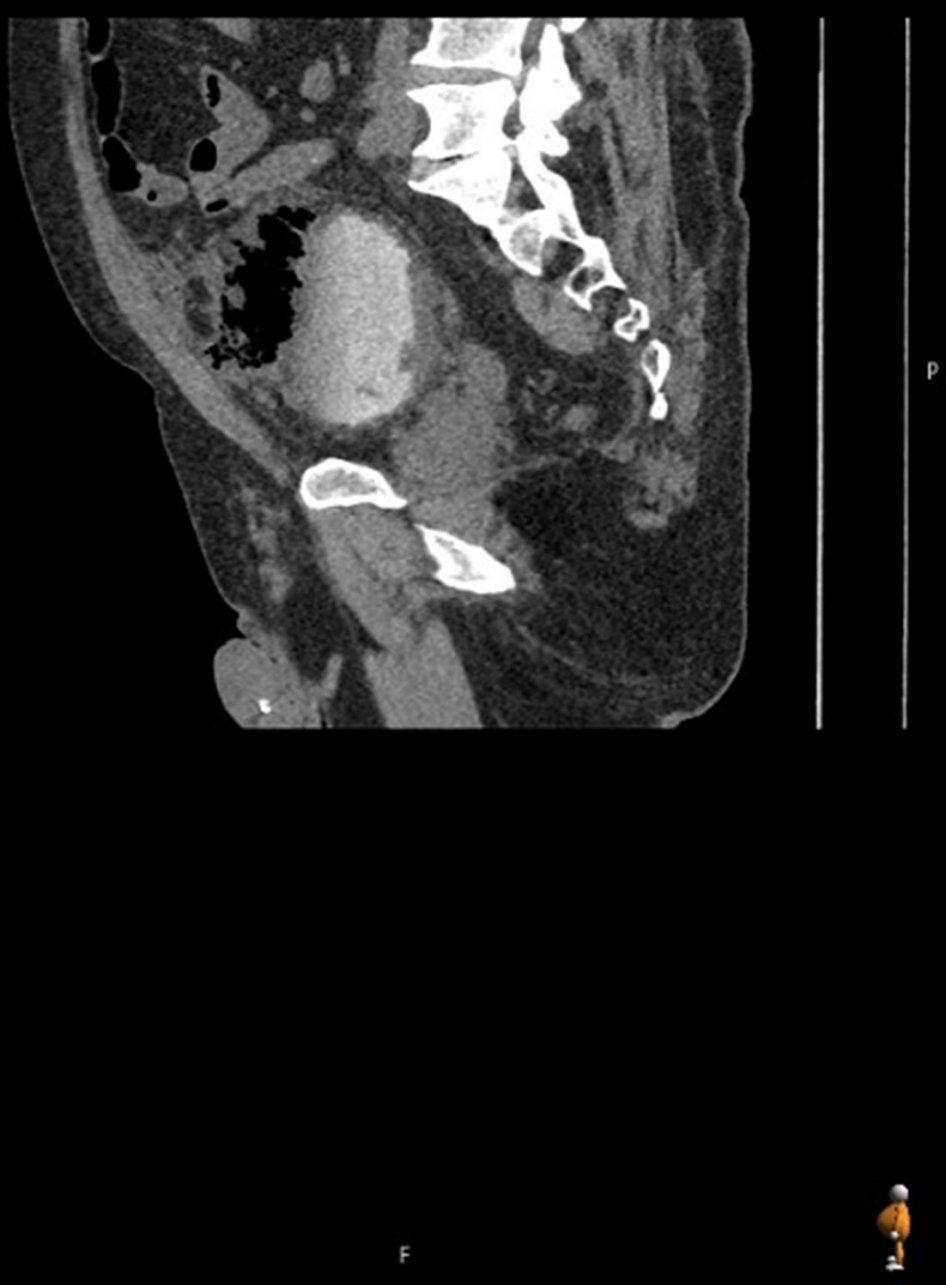
Second sagittal delayed phase CT (Day 8 of admission), on soft tissue window setting, demonstrating a large bladder diverticulum containing a gas–fluid level however the previously demonstrated intramural gas has resolved; extraluminal gas is seen in the anterior antidependent regions of the pelvis/lower abdomen indicative of perforation, the volume of which has reduced compared to the earlier CT examination.

On Day 8 of admission, a second CT scan was performed (Figures 8–10), which showed a reduction in the degree of infectious/inflammatory changes relating to the bladder diverticulum and a reduced volume of extraperitoneal free gas. Following medical therapy, the patient responded well, and the inflammatory markers were approaching normal (C-reactive protein: 17, white cell count: 11.4, neutrophils: 11.0). The patient’s renal function had normalised by the time of discharge (urea 5.7, creatinine 94).

**Figure 11.**
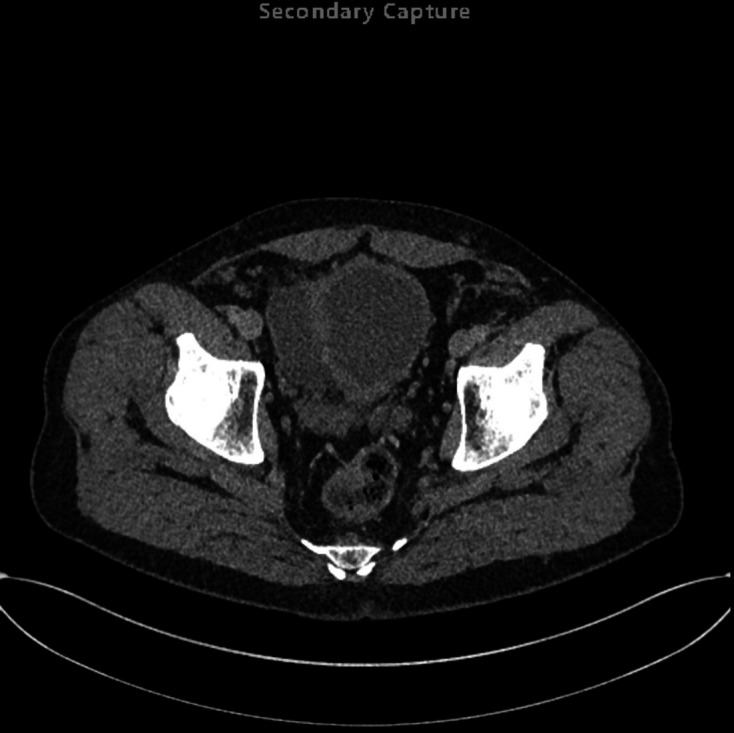
Third axial delayed phase CT (3 months following admission), on soft tissue window setting, demonstrating a chronically thick-walled bladder; to the anatomical right side of the bladder there is a large fluid-filled bladder diverticulum however the previously demonstrated intraluminal and intramural gas has resolved; the previously demonstrated extraluminal gas has also resolved.

**Figure 12.**
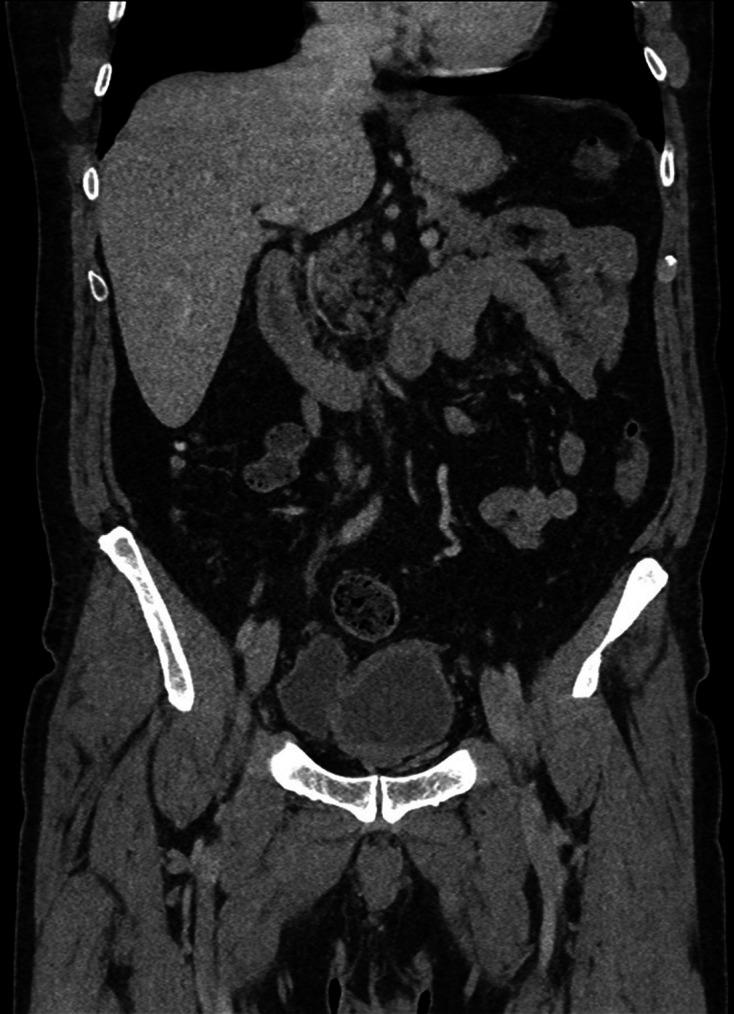
Third coronal delayed phase CT (3 months following admission), on soft tissue window setting, demonstrating a chronically thick-walled bladder; to the anatomical right side of the bladder there is a large fluid-filled bladder diverticulum, however, the previously demonstrated intraluminal and intramural gas has resolved; the previously demonstrated extraluminal gas has also resolved.

**Figure 13.**
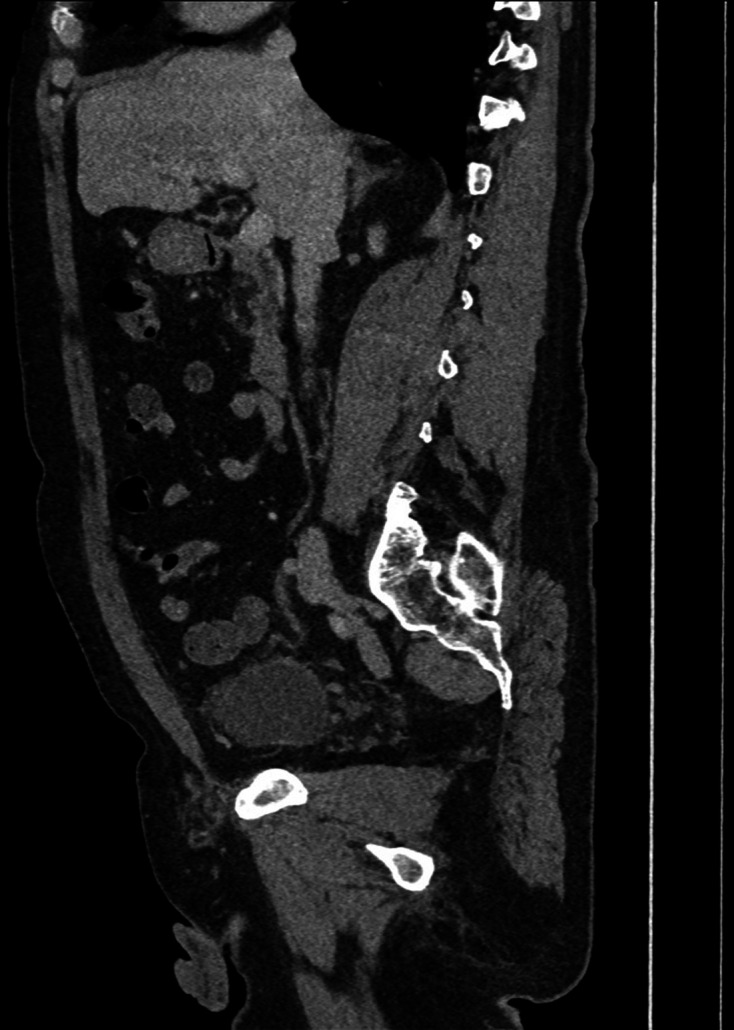
Third coronal delayed phase CT (3 months following admission), on soft tissue window setting, demonstrating a large fluid-filled bladder diverticulum, however, the previously demonstrated intraluminal and intramural gas has resolved; the previously demonstrated extraluminal gas has also resolved.

3 months following admission, a third CT scan was performed (Figures 11–13), which demonstrated chronic bladder wall thickening but no acute inflammatory changes to suggest an active infection and no gas in the intraluminal, intramural or extraluminal spaces.

## Differential diganosis

The patient was treated for emphysematous cystitis involving a perforated bladder diverticulum.

Traumatic perforation following repeated catheterisation was considered a possibility but it was felt that the perforation was more likely due to the underlying infectious/inflammatory process that had weakened the diverticulum wall allowing gas-producing microorganisms to leak gas into the abdominal cavity.

Due to the volume of free abdominal air, gastrointestinal perforation was also considered but appearances of the bowel were shown to be normal on CT allowing this possibility to be dismissed.

## Treatment

Emphysematous cystitis was managed with intravenous amikacin and co-amoxiclav; although no antimicrobial sensitivities were identified the patient responded well to empirical treatment. The acute kidney injury was treated with intravenous fluid and ramipril was withheld due to its nephrotoxicity. After the presence of extraperitoneal free gas was confirmed on CT, urological surgical intervention was considered but the patient had responded well to medical therapy, so surgical treatment was not indicated. The patient’s acute kidney injury also responded well to conservative management.

## Outcome and follow-up

The patient’s clinical condition, laboratory markers and radiological findings all showed significant interval improvement to medical therapy. Although the patient had no known history of diabetes mellitus, laboratory findings suggested there may have been a degree of underlying glucose intolerance, which would have contributed to an increased risk in the patient developing emphysematous cystitis. After 11 days of in-patient care, the patient was discharged with oral antibiotics and long-term urinary catheter.

## Discussion

Due to its rarity, there is little established knowledge about the mechanisms of emphysematous cystitis, although plausible theories have been suggested. Such theories usually point to increased glucose in the diabetic patients’ tissues, allowing fermentation to take place which lowers pH, resulting in carbon dioxide production.^7^ The glucose-rich tissue theory, however, does little to account for the infections that take place in non-diabetics, therefore alternatively it has been suggested that urinary albumin may act as a substrate for gas-forming pathogens.^8^

There have been numerous case reports of emphysematous cystitis, most highlight the difficulty in diagnosis and the low threshold of suspicion required to diagnose promptly. Overall mortality rates from emphysematous cystitis are reported to be 7%,^5^ however, this is likely an overestimate due to its relative underdiagnosis. Somewhat rarer, are cases that combine bladder perforation with emphysematous cystitis.^4^^9^ In all cases, the emphysematous cystitis and coincident bladder perforation are diagnosed radiologically, although treatment differs depending on the severity and location of the lesion. To the best of our knowledge, no other cases of atraumatic bladder diverticulum perforation secondary to emphysematous cystitis have been reported in the literature.

Atraumatic bladder diverticulum perforation remains a controversial clinical entity, with its specific mechanisms not understood and few cases reported in the literature.^10^ Bladder diverticulum walls are weaker by nature (only lined with serosa and adventitia), and are therefore more prone to perforation due to retention and tissue infection. Emphysematous cystitis is more likely in the diabetic patient and the resultant pressure from the gas produced, in addition to the tissue inflammation present during infection, makes tissues more friable and bladder diverticulum perforation therefore more likely. CT is the key tool in the diagnostic work up for emphysematous cystitis and bladder perforation, its widespread use in modern medicine likely accounting for the large increase in emphysematous cystitis diagnoses.^5^

Published case reports offer examples of effective treatment regimens for this condition. The generally adhered to treatment protocol of emphysematous cystitis, after a prompt diagnosis, revolves around broad spectrum intravenous antibiotics, tight glycaemic control, good bladder drainage and subsequent surgery, if the aforementioned are ineffective.^5^ The rationale for the above treatment: to limit any element of sepsis, to give the bladder rest to best allow tissues to heal and good diabetic control to reduce pathogenesis within the insult.

This is a unique case report highlighting a hitherto unreported bladder diverticulum perforation. Given the high risks associated with atraumatic bladder perforation, it is valuable to publish cases to enable fellow clinicians to have a higher index of suspicion when assessing at risk patients with similar symptoms.

## Learning points/take home messages

- A bladder diverticulum can become a focus for cystitis; secondary perforation is a rare but potentially fatal complication.
- Risk factors include diabetes mellitus, advanced age, alcoholism, urinary stasis and female sex.
- Early diagnosis will allow early medical management, which will reduce morbidity and mortality.
